# Lymphatic endothelial sphingosine 1-phosphate receptor 1 enhances macrophage clearance via lymphatic system following myocardial infarction

**DOI:** 10.3389/fcvm.2022.872102

**Published:** 2022-08-08

**Authors:** Qinyu Li, Caixia Zhou, Kang Zhao, Yunhao Duan, Jinnan Yue, Xiuxiang Liu, Jinjin Wu, Shengqiong Deng

**Affiliations:** ^1^Postgraduate Training Base in Shanghai Gongli Hospital, Ningxia Medical University, Ningxia, China; ^2^Key Laboratory of Arrhythmias of the Ministry of Education of China, Research Center for Translational Medicine, Shanghai East Hospital, Tongji University School of Medicine, Shanghai, China; ^3^Cardiovascular Department, Shanghai Children’s Medical Center, Shanghai Jiao Tong University School of Medicine, Shanghai, China; ^4^Department of Clinical Laboratory, School of Medicine, Gongli Hospital, Shanghai University, Shanghai Health Commission Key Lab of Artificial Intelligence (AI)-Based Management of Inflammation and Chronic Diseases, Shanghai, China

**Keywords:** sphingosine 1-phosphate receptor 1, lymphatic endothelial cells, myocardial infarction, macrophages, lymphatic system

## Abstract

Lymphatic endothelial cell homeostasis plays important roles in normal physiological cardiac functions, and its dysfunction significantly influences pathological cardiac remodeling after myocardial infarction (MI). Our results revealed that sphingosine 1-phosphate receptor 1 (S1pr1) expression in cardiac lymphatic endothelial cells (LECs) was sharply changed after MI. It has been shown that S1pr1 tightly controlled LEC functions and homeostasis. We thus hypothesized that lymphatic endothelial S1pr1 might be involved in post-MI cardiac remodeling. We generated LEC-conditional S1pr1 transgenic mice, in which S1pr1 expression was reduced in cardiac LECs. We performed the left anterior descending coronary artery (LAD) ligation operation to induce MI in these mice. Cardiac functions and remodeling were examined by echocardiography analysis and serial histological analysis. Meanwhile, we performed adoptive cell transfer experiments to monitor macrophage trafficking in post-MI myocardium and their draining lymphatic system. Furthermore, *in vitro* cell culture experiments and mechanism studies were undertaken to uncover the molecular mechanism by which LEC-S1pr1 regulated cardiac inflammation and remodeling after MI. Our results showed that S1pr1 expression significantly decreased in cardiac LECs after MI. Our *in vivo* experiments showed that the reduced expression of LEC-S1pr1 deteriorated cardiac function and worsened pathological cardiac remodeling after MI. Our further results demonstrated that the reduced expression of LEC-S1pr1 did not influence macrophage infiltration in an early inflammatory phase of MI, but significantly affected macrophages clearance in the later phase of MI via afferent cardiac lymphatics, and thus influenced inflammatory responses and cardiac outcome after MI. Further study showed that S1P/S1pr1 activated ERK signaling pathway and enhanced CCL2 expression, which promoted macrophage trafficking in a paracrine manner. This study reveals that cardiac lymphatic endothelial cells tightly control macrophage trafficking via lymphatic vessels in injured hearts via S1P/S1pr1/ERK/CCL2 pathway and thus regulate post-MI immune modulation and heart repair. This study highlights the importance of cardiac lymphatic vessel system in orchestrating post-MI immune responses and cardiac remodeling by regulating macrophage transit in injured hearts. Our finding implies that a feasible modulation of S1pr1 signaling in LECs might provide a promising target to resolve excessive inflammation and to ameliorate adverse cardiac remodeling after MI.

## Introduction

Myocardial infarction (MI) remains a leading cause of morbidity and mortality worldwide (1, 2). Although significant advances and development in the treatment of myocardial infarction have resulted in a progressive decline in long-term risk of sudden death, the prevalence of heart failure after MI is increasing globally (1). Heart failure is accompanied by pathological ventricular remodeling, resulting in ventricular diastolic and systolic dysfunction (3). Therapeutic strategies to improve post-MI cardiac remodeling have been proposed as means of preventing the progression of heart failure (4). Therefore, a more comprehensive understanding of molecular mechanisms of pathological cardiac remodeling is urgently required to establish a novel therapeutic approach for heart failure.

The lymphatic vasculature pervades almost all tissues and organs, and plays an important role in the maintenance of interstitial fluid homeostasis and immune surveillance (5). Previous clinical investigations reported that lymphatic vascular remodeling displayed in coronary heart disease (CHD) and heart failure (HF) patients (6–8). In preclinic experiments, cardiac lymphangiogenesis occurs in animal models of MI or heart transplantation (6, 9–12). Other studies of post-MI hearts have shown that cardiac lymphatic vasculature was involved in the regulation of immune cell trafficking in ischemic myocardium and reduced cardiac inflammation (13). It has been previously reported that allograft lymphangiogenesis and up-regulation of VEGFC and VEGFR3 expression were observed in heart transplantation animal experiments (11, 12). Lymphatic system was activated in the allograft hearts upon ischemia/reperfusion injury to the graft before cardiac transplantation (12). Previous investigations showed that stimulation of cardiac lymphatic vessels with VEGF-C improved resolution of the acute inflammatory response after MI by trafficking immune cells to draining mediastinal lymph nodes (MLNs) (13). These findings highlight the important role of lymphatic endothelium in the regulation of immune cell clearance and cardiac inflammation. Understanding how cardiac lymphatics limit myocardial inflammation after MI might provide a novel strategy to improve post-MI pathological ventricular remodeling and restore cardiac function.

Recent studies revealed that the expression of S1PR1/S1pr1 was dysregulated in both the ischemic human hearts and post-MI murine hearts, indicating that S1P/S1pr1 might be involved in pathological cardiac remodeling after MI [Gowda et al. (14)]. It has been well known that S1P tightly regulated endothelial cell functions via S1pr1 (3). S1pr1 plays an important role in vascular integrity and angiogenesis (3, 15, 16). Loss of EC-S1pr1 results in the enhanced endothelial cell sprouting and formation of ectopic vessel branches, suggesting that EC-S1pr1 acts as a vascular-intrinsic stabilization mechanism during vascular development (15, 16). Previous studies revealed that EC-S1pr1 displayed a similar effect on sprouting angiogenesis in tumor angiogenesis as in developmental angiogenesis (17). During embryogenesis, lymphatic endothelial cells are derived mostly from blood vascular endothelial cells (18). Therefore, lymphatic endothelium shares many molecular characteristics of blood vascular endothelium, including the expression of S1pr1 (18). Geng X et al. reported that S1pr1 was involved in the regulation of lymphatic vascular quiescence and maturation during development (19). The specific loss of S1pr1 in LECs resulted in lymphatic vascular hyper-sprouting and hyper-branching in mouse embryos (19), indicating that LEC-S1pr1 plays an important role in the maintenance of lymphatic vasculature homeostasis during embryogenesis. However, the role of LEC-S1pr1 in the regulation of cardiac function during pathological cardiac remodeling after MI is completely unknown.

Here we reported that S1pr1 expression significantly decreased in cardiac LECs after MI. We established heterozygous *Lyve1-Cre-S1pr1*^flox/wt^** mice, in which *S1pr1* expression levels were reduced in cardiac LECs. Our *in vivo* experiments showed that the reduced expression of LEC-S1pr1 deteriorated cardiac function and worsened pathological cardiac remodeling after MI. Our further results demonstrated that the reduced expression of LEC-S1pr1 did not influence macrophage infiltration in an early inflammatory phase of MI, but significantly affected macrophage clearance in the later phase of MI via afferent cardiac lymphatics. The mechanism study showed that S1P/S1pr1 activated ERK signaling pathway and enhanced CCL2 expression, which promoted macrophage trafficking in a paracrine manner. This study reveals that cardiac lymphatic endothelial cells tightly control macrophage trafficking via lymphatic vessels in injured hearts via S1P/S1pr1/ERK/CCL2 pathway and thus promote post-MI immune modulation and heart repair.

Taken together, this study highlights the importance of cardiac lymphatic vessel system in orchestrating post-MI immune responses and cardiac remodeling by regulating trafficking of macrophages in injured hearts. Our studies demonstrate that S1P/S1pr1/CCL2 praxis in LECs plays an important role in the regulation of post-MI macrophage trafficking via the cardiac lymphatic system. This finding implies that a feasible modulation of S1pr1 signaling in LECs might provide a promising target to resolve excessive inflammation and to ameliorate adverse cardiac remodeling after MI.

## Materials and methods

### Generation of lymphatic endothelial cell-specific *S1pr1* allele deletion mice

The conditional *S1pr1* knock-out (*S1pr1^flox/flox^*) mice were crossed with *Lyve1* promoter-driven *Cre* line (*Lyve1-Cre*) to generate lymphatic endothelial-specific *S1pr1* allele deletion mice (3, 19). 8-week-old male littermates with *C57BL/6J* background were applied in all experiments. All animal procedures were operated in accordance with the Institutional Animal Care and Use of Laboratory Animals approved by the Tongji University Institutional Animal Care and Use Committee with license number TJLAC-0126-026.

### Induction of myocardial infarction animal models

The operation conformed to the “Guide for the Care and Use of Laboratory Animals” published by the US National Institutes of Health (NIH Publication No. 85-23, 1996, revised 2011). Briefly, mice were anesthetized with 1% pentobarbital sodium (40 mg/kg, i.p.), and mechanically ventilated (isoflurane 1–2% vol/vol) with a rodent respirator device. Mice chest cavity was opened with a left thoracotomy to expose their hearts. We ligated the left anterior descending coronary artery (LAD) with an 8-0 silk suture under the stereomicroscope, as previously reported (20). Complete occlusion of the vessel was confirmed by the presence of myocardial bleaching in the LAD coronary artery perfusion area.

### Echocardiography analysis

Echocardiography was applied to evaluate the cardiac geometry and systolic function as previously reported (21). A Visual Sonics high-resolution Vevo2100 ultrasound system (Visual Sonics Inc., Canada) with a 30-MHz linear array ultrasound transducer (MS-400, Visual Sonics In.) was used. Briefly, mice were anesthetized with 2.0% isoflurane till the heart rate stabilized at 400–500 b.p.m. (beats per minute). Parasternal long-axis images were obtained in B-mode with an appropriate position of the scan head and the maximum LV length identified. In this view, the M-mode cursor was placed perpendicular to the maximum LV dimension in end-diastole and end-systole.

### Histology

The hearts were collected 28 days after MI. The hearts were fixed, dehydrated, embedded, and sectioned into 5 μm-thick pieces. The tissue sections were stained with hematoxylin & eosin (H&E) and Masson’s trichrome. Immunostaining was performed on cryostat 8 μm-thick sections by using various antibodies, including rabbit anti-mouse lymphatic vessel endothelial hyaluronan receptor (LYVE-1, Abcam, #ab14917), Anti-F4/80(Abcam, #ab6640), Anti-Ly6g (Abcam, #ab25377) Anti-Wheat Germ Agglutinin (WGA)-alexa488 (Invitrogen, #W11262), biotinylated-isolectin B4 antibody (IB4, Vector Laboratories, B-1205) and their corresponding secondary antibodies. Nuclei were stained with DAPI (Sigma, #D9542). To evaluate capillary vessel density, the relative capillary density by calculation of the isolectin B4 positive capillary numbers per high-power microscopic field was calculated in IB4-staining tissue sections.

### Cell culture

Human lymphatic endothelial cells (HLECs, ATCC) were cultivated in EGM2 (Endothelial cell growth medium 2, PromoCell) and cells within 8th passage were used for *in vitro* experiments. HLECs were infected with lentiviral S1PR1 over-expressing, shRNA of S1PR1 or scramble control vectors.

Boyden chamber and scratch wound healing assay were applied for assessing cell migration. Briefly, in transwell assays, LECs were placed on the upper layer of the Boyden chamber (Falcon 353097) assay, and culture medium EGM2 was placed below the cell-permeable membrane. After 6-h incubation, the cells that have migrated through the membrane were stained by crystal violet solution and counted. In scratch wound healing assay, HLECs were plated in plates to reach 90% confluence. A vertical wound scratch was generated and HLECs were then cultured with FBS-reduced EGM2 medium. At designated times, the wound of HLECs culture was imaged and assessed wound closure rate.

MTT assay was used for cell proliferation assay. Briefly, HLECs were placed in a flat-bottom 96-well cell culture plates for 48 h. 10 μl MTT (5 ng/ml) solution (Sigma) was then added to each well followed by 4-h incubation at 37°C, and the media was replaced with 150 ml DMSO. The cell culture plates were shaken for 10 min at room temperature, and then monitored by a microplate reader (Bio-Rad) at 490 nm for measurement of the absorbance in each well.

For tube formation assay, the Matrigel with a volume of 50 μl was added to each well of a 96-well cell culture plate on ice, and then the plate was incubated at 37°C for 1 h in order to allow matrigel to solidify, as previously reported (22). HLECs were resuspended in the desired culture medium at 1 × 10^5^ cells/ml. 200 μl of cell suspension (5 × 10^4^ cells) were plated into each well onto the solidified matrigel gel. The assay plate was incubated at 37°C, 5% CO_2_ for 4–8 h, and tube formation was observed by a phase-contrast light microscope. The total tube length and branch points were measured using ImageJ software.

For isolation of primary lymphatic endothelial cells, 8-week-old mice were sacrificed and their hearts were isolated aseptically and minced finely, and then digested in collagenase I (1 mg/ml, Sigma Aldrich, #C6885), DNase I (60 U/ml, Themo Fisher, #EN0521) at 37°C at 120 rpm for 40 min. After passing through a 70-μm nylon strainer, the single cell suspension of myocardium was incubated with CD11b microbeads (Mitenyi Biotec, #130-049-601) to deplete monocyte/macrophages, and the washed cells without CD11b microbeads-bound cells were further incubated in 0.1% bovine serum albumin/PBS with anti-Lyve-1 antibody-conjugated Dynabeads (Invitrogen, Carlsbad, CA), and rotated for 20 min. Primary lymphatic endothelial cells were then separated by a magnetic separator and used for further quantitative analysis of gene expression by real-time quantitative polymerase chain reaction (PCR). Using this new cell separation strategy, we achieved the purity of LECs more than 95% without monocyte/macrophage contamination, as shown in the below representative images of flow cytometric analysis (Supplementary Figures 1A–C).

For co-culture experiment, the supernatants of HLECs transfect with S1PR1-overexpressing lentivirus, S1PR1 shRNA lentivirus and their corresponding empty lentivirus were collected. THP-1 cell lines were placed on the upper layer of Boyden chamber, and the supernatants of different groups were placed below the cell permeable membrane. After 6-h incubation, THP-1 that have migrated through the membrane were stained by crystal violet solution and counted.

### Flow cytometric analysis

For Lymphatic endothelial cell and monocyte/macrophage staining, cells were stained for PE anti-mouse/human CD11b (Biolegend, #101208), Pacific Blue™ anti-mouse CD45 (Biolegend, #103126), APC anti-mouse F4/80 (Biolegend, #123116), Lyve-1 (Abcam, #ab14917), Ly6C-PE (Thermo Fisher, #12593282), Alexa Fluor™ 488 goat anti-rabbit IgG (Thermo Fisher Scientific, #A11034). Data were recorded with an LSR II flow cytometer (BD Biosciences) and analyzed with FlowJo (Version 9.0).

### Ribonucleic acid Extraction, ribonucleic acid microarray and real-time polymerase chain reaction for gene expression

Total RNA from hearts or LECs was extracted by Trizol (Invitrogen) following the manufacturer’s instructions and used for RT-PCR (the primer list was listed in Table 1) or high-throughput Gene Expression microarray (Agilent Technology). RNA quantity and quality were measured by NanoDrop ND-1000.

**TABLE 1:** The primer list for real-time quantitative polymerase chain reaction analysis of mRNA expression levels.

*hS1PR1 F*	GCAGTACAGAATGACGATGGAG
*hS1PR1 R*	GCCTCTTCCTGCTAATCAGCG
*hCCL2 F*	CCTCTGCACTGAGATCTTCCTAT
*hCCL2 R*	TCGCTCAGCCAGATGCAA
*hCCL3 F*	CCAGTTCTCTGCATCACTTGC
*hCCL3 R*	GAATCTGCCGGGAGGTGTAG
*hCCL6 F*	ATCCTTGTGGCTGTCCTTGG
*hCCL6 R*	TGAAGAAGTGTCTTGAAAGCCTTG
*hGAPDH F*	GGAGCGAGATCCCTCCAAAAT
*hGAPDH R*	GGCTGTTGTCATACTTCTCATGG
*mS1pr1F*	ATGGTGTCCACTAGCATCCC
*mS1pr1R*	CGATGTTCAACTTGCCTGTGTAG
*mS1pr2 F*	TGTTGCTGGTCCTCAGACGCTAG
*mS1pr2 R*	CCAGAAATGTCGGTGATGTAGGC
*mS1pr3F*	ACTCTCCGGGAACATTACGAT
*mS1pr3R*	CCAAGACGATGAAGCTACAGG
*mS1pr4F*	CTGGCTACTGGCAGCTATCC
*mS1pr4R*	AGACCACCACACAAAAGAGCA
*mS1pr5F*	TGGCTAACTCGCTGCTGAATC
*mS1pr5R*	TCGCTGCAAGCTGTTGGAG
*mLyve1 F*	CTCGTGCAAGACCTTTCCATT
*mLyve1 R*	GCCTCGTTGGCTTCTGTGAA
*mCcl2 F*	TAAAAACCTGGATCGGAACCAAA
*mCcl2 R*	GCATTAGCTTCAGATTTACGGGT
*mGapdh F*	TGGCCTTCCGTGTTCCTAC
*mGapdh R*	GAGTTGCTGTTGAAGTCGCA

### Western blot

The total cell proteins of HLECs were extracted and resolved by 10% SDS-PAGE gels. Proteins were electrophoretically transferred to PVDF membranes (Millipore, United States) for western blotting. The membranes were blocked in 5% skim milk for 1 h at room temperature, and then incubated with the corresponding primary antibodies overnight at 4 °C with rotation. The primary antibodies included Anti-ERK1/2 (CST, #4695) and phospho-ERK1/2 (CST, #4370), Anti-GAPDH (CST, #2118). After incubation with the corresponding peroxidase-conjugated secondary antibody, the signals were visualized using a chemiluminescence kit (CST).

### Adoptive cell transfer

We performed adoptive cell transfer experiments, as previously reported (13). Briefly, monocytes were isolated from the spleen of ubiquitous EGFP expressing transgenic mice. The spleen was removed and then disrupted with the blunt end of a sterile syringe. The spleen cell suspension was subsequently incubated in red cell lysis buffer (Roche, #11814389001) for 10 min at room temperature, and then was passed through a 70-μm cell strainer. After passing through a 70-μm nylon strainer, the single cell suspension of spleen was incubated in 0.25% bovine serum albumin/PBS. Monocyte enrichment was achieved by EasySep Mouse Monocyte Enrichment Kit (STEMCELL Technologies) according to the manufacturer’s protocol. The cells were then spun down and resuspended in PBS buffer. After LAD operation, each EGFP^–^ recipient mouse received 100,000 EGFP^+^ monocytes were cells by multiple intramyocardial injections at the time of LAD operation. The hearts and their draining mediastinal lymph nodes (MLNs) were isolated for histological analysis.

### Statistics

All continuous data were represented as means ± standard error of mean (S.E.M.) for at least three independent assays unless otherwise noted. Student’s *t*-test was used for comparisons between two groups. One-way or two-way ANOVA analysis followed by Tukey was applied in multiple comparison analyses. *P* < 0.05 was considered as statistical significance in this study. All data were checked for normality and equal variance before using parametric tests. All analyses were performed with SPSS 11.0 (SPSS. Inc) for Windows.

## Results

### The expression of sphingosine 1-phosphate receptor 1 is down-regulated in lymphatic endothelial cells after acute myocardial infarction

**FIGURE 1:**
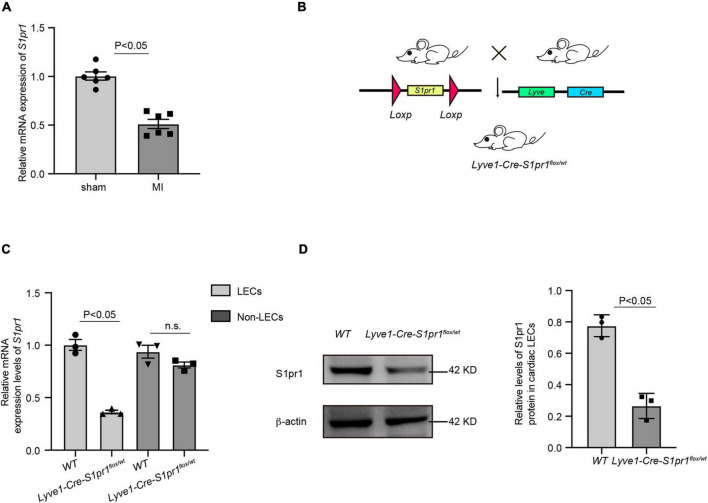
The expression of S1pr1 was down-regulated in cardiac lymphatic endothelial cells. **(A)** Relative mRNA expression levels of S1pr1 in cardiac LECs of mice at 3 days after sham or LAD operation (*n* = 6). **(B)** Schematic diagram of generation of mice with LEC-specific loss of *S1pr1* allele. **(C)** Relative mRNA expression levels of S1pr1 in cardiac LECs of *WT* and *Lyve1-Cre-S1pr1*^flox/wt^** mice (*n* = 3). **(D)** Western-blotting analysis of S1pr1 expression in cardiac LECs from the indicated groups, with its quantification (*n* = 3). Data are mean ± S.E.M.

It has been shown that lymphatic system homeostasis played an important role in cardiac remodeling after myocardial infarction (MI) (18). To investigate whether sphingosine 1-phosphate receptor 1 (S1pr1) is expressed in cardiac LECs and whether LEC-expressing S1pr1 is involved in the regulation of cardiac remodeling after MI, we measured the S1pr1 mRNA expression in cardiac LECs after MI by quantitative RT-PCR. Our results showed that S1pr1 expression was the domain S1P receptor subtype in cardiac lymphatic endothelial cells and significantly decreased at 3 days after MI (Figure 1A and Supplementary Figures 2A–C). Because previous investigations demonstrated that S1pr1 was highly expressed in lymphatic endothelial cells (LECs) and tightly controlled lymphatic vessel functions (19), we hypothesized that LEC-S1pr1 might influence cardiac remodeling after MI. To further determine the effects of LEC-S1pr1 on MI, we crossed the *Lyve1-Cre* mouse line with *S1pr1^flox/flox^* mice (Figure 1B) to specifically delete *S1pr1* allele in lymphatic endothelial-specific. In consistence with the previous report (19), homozygous *Lyve1-Cre-S1pr1*^flox/flox^** displayed perinatal lethality and we hardly obtained adult *Lyve1-Cre-S1pr1*^flox/flox^** mice. We next tested whether the expression of S1pr1 in LECs was reduced in heterozygous *Lyve1-Cre-S1pr1*^flox/wt^** mice. As shown by RT-qPCR, S1pr1 mRNA levels were significantly reduced in cardiac LECs of heterozygous *Lyve1-Cre-S1pr1*^flox/wt^** mice, but not in non-LEC cardiac cells (Figure 1C). Correspondingly, western-blotting showed that *S1pr1* protein levels were significantly decreased in LECs of heterozygous *Lyve1-Cre-S1pr1*^flox/wt^** mice (Figure 1D).

### The reduced expression of LEC-S1pr1 deteriorates cardiac dysfunction after acute myocardial infarction

**FIGURE 2:**
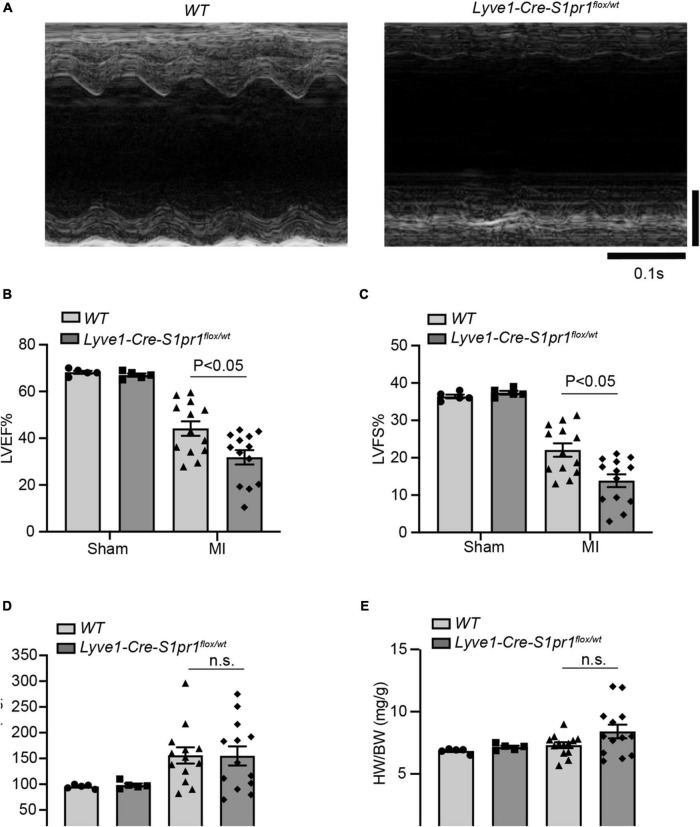
The reduced expression of LEC-S1pr1 deteriorates cardiac function after acute myocardial infarction. **(A–D)** Representative images of echocardiography of post-MI hearts in *WT* and *Lyve1-Cre-S1pr1*^flox/wt^** mice **(A)** with quantification of left ventricle ejection fraction (LVEF%), left ventricle fractional shortening (LVFS%), and LV mass (mg) (**B,D**) (*n* = 3). **(E)** The ratio of heart weight to bodyweight of the indicated groups (HW/BW, mg/g) (*n* = 3). Data are mean ± S.E.M. n.s., no statistical significance.

We next performed left anterior descending coronary artery (LAD) ligation operation to induce acute myocardial infarction in *Lyve1-Cre-S1pr1*^flox/wt^** mice. We assessed cardiac function by echocardiography at 28 days after MI. Although reduced *S1pr1* expression in LECs didn’t influence heart functions without injury, a reduction in LEC-*S1pr1* expression led to a significant reduction in left ventricular ejection fraction (LVEF%) and left ventricular fractional shortening (LVFS%) after MI, compared with control *WT* littermates (Figures 2A–C). Our further data showed that the reduced LEC-S1pr1 expression didn’t influence cardiac hypertrophy, as shown by LV mass and the ratio of heart weight to body weight (Figures 2D–E). The data demonstrate an important role of LEC-S1pr1 in regulating cardiac dysfunction after acute MI.

### The reduced expression of LEC-S1pr1 aggravates post-mI pathological cardiac remodeling

**FIGURE 3:**
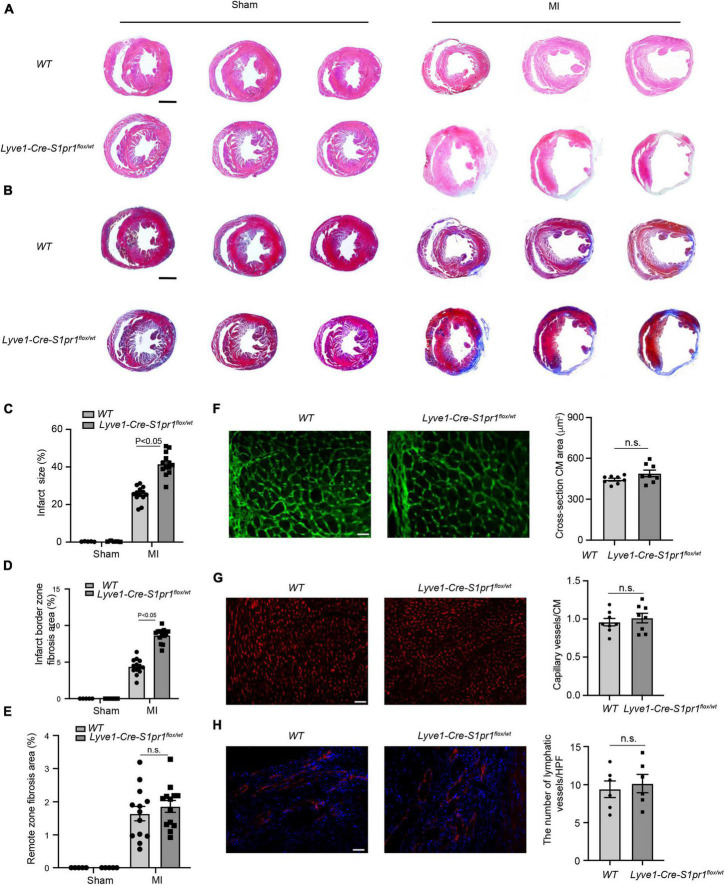
The reduced expression of LEC-S1pr1 aggravates post-MI pathological cardiac remodeling. **(A)** Representative images of H&E staining of serial sections from hearts in *WT* and *Lyve1-Cre-S1pr1*^flox/wt^** mice at 28 days after MI (*n* = 13). **(B–E)** Representative images of Masson’s Trichrome staining of serial sections from hearts in *WT* and *Lyve1-Cre-S1pr1*^flox/wt^** mice at 28 days after MI (**B**), with quantification of the percentage of infarct size and cardiac fibrosis in left ventricle myocardium (**C–E**) (*n* = 13). **(F)** Representative images of WGA staining of hearts in *WT* and *Lyve1-Cre-S1pr1*^flox/wt^** mice at 28 days after MI, with quantification of the cross-section cardiomyocyte area in hearts (*n* = 8). **(G)** Representative images of isolectin-B4 staining of hearts in *WT* and *Lyve1-Cre-S1pr1*^flox/wt^** mice at 28 days after MI, with quantification of capillary density in post-MI myocardium (*n* = 8). **(H)** Representative images of Lyve-1 (LEC marker) staining of hearts in *WT* and *Lyve1-Cre-S1pr1*^flox/wt^** mice at 28 days after MI, with quantification of capillary **l**ymphatic density in post-MI myocardium (*n* = 6). Data are mean ± S.E.M. Scale Bars: **(A,B)**, 2 mm; **(F–H)**, 50 μm; n.s., no statistical significance.

We next performed histological analysis to investigate the effects of LEC-S1pr1 on cardiac remodeling after MI. *Lyve1-Cre-S1pr1*^flox/wt^** mice displayed larger infarct size (Figure 3A) as shown by H&E staining and greater fibrotic scar size as shown by Masson’s Trichrome staining (Figures 3B–E). Our data showed that *Lyve1-Cre-S1pr1*^flox/wt^** mice exhibited a similar cardiomyocyte size after MI as *WT* littermates (Figure 3F). A similar blood vessel capillary density in the infarct border area was observed in *Lyve1-Cre-S1pr1*^flox/wt^** mice compared with *WT* mice (Figure 3G). Immunostaining of Lyve1 showed that there were no differences in lymphatic vessels between *Lyve1-Cre-S1pr1*^flox/wt^** mice and WT littermates, indicating that a reduction of S1pr1 expression in LECs doesn’t influence lymphatic vessel formation in hearts (Figure 3H). Moreover, the reduced expression of S1pr1 didn’t influence cell proliferation in LECs in 7 days post-MI hearts (Supplementary Figure 3). These data demonstrated that the reduced LEC-S1pr1 expression worsened post-MI pathological cardiac remodeling without any significant influences on the lymphatic vessel formation in post-MI hearts.

### Sphingosine 1-phosphate receptor 1 regulates lymphatic endothelial cell migration, proliferation, and angiogenic activity

**FIGURE 4:**
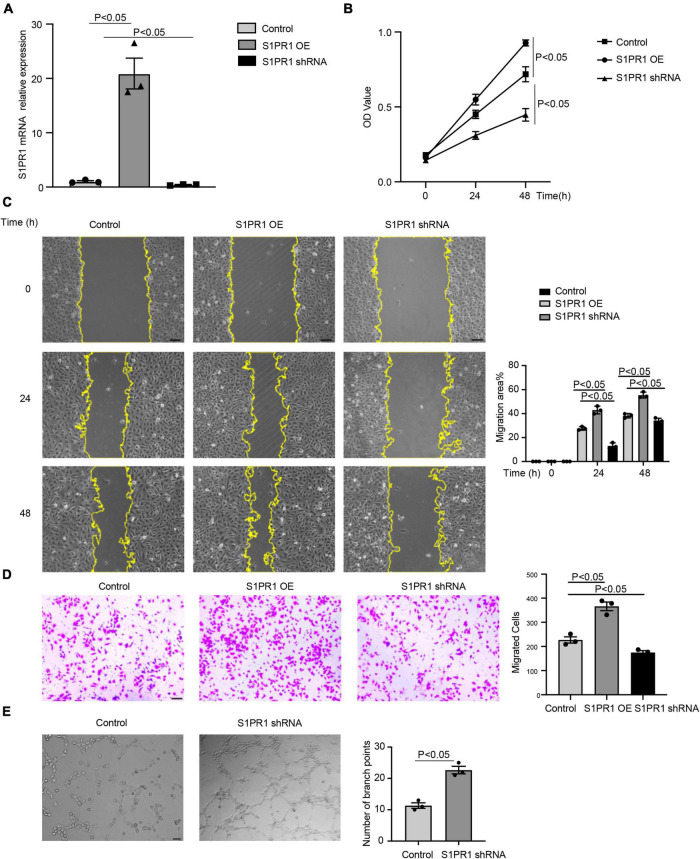
S1pr1 regulates lymphatic endothelial cell migration, proliferation, and angiogenic activity. **(A)** RT-qPCR analysis of S1PR1 mRNA levels in LECs of the indicated group (*n* = 3). **(B)** MTT assay showed the proliferation of cells of the indicated group (*n* = 3). **(C)** Scratch wound healing assay showed the cell migration, with quantification of cell migration area (%) of the indicated group (*n* = 3). **(D)** Boyden chamber assay showed the cell migration of cells of the indicated group, with quantification of migrated cells of the indicated group (*n* = 3). **(E)** Tube formation assay showed S1PR1 shRNA promoted LEC tube formation, with quantification of the number of branch points (*n* = 3). Control, scramble RNA lentivirus. OE, S1PR1-overexpressing lentivirus. shRNA, S1PR1 shRNA lentivirus. Data are mean ± S.E.M. Scale Bars: (**C–E)**, 100 μm.

To better understand the effects of S1pr1 in LECs, cell migration and cell proliferation were performed in human lymphatic endothelial cells (HLECs). Lentivirus carrying S1PR1 (S1PR1 OE) or S1PR1 shRNA was generated to achieve elevated or reduced expression of S1PR1 in HLECs, respectively. Overexpression of S1PR1 in HLECs markedly increased cell migration and cell proliferation as shown by the transwell chemotactic assay and scratch wound healing assay and MTT assay, respectively, and the reduced S1PR1 expression significantly inhibited LEC migration and proliferation (Figures 4A–D), demonstrating that S1pr1 enhances lymphatic endothelial cell migration and proliferation. We next analyzed the effect of S1PR1 on *in vitro* angiogenic activity of HLECs using tube formation assay. The higher angiogenic tube formation was observed in HLEC-expressing S1PR1 shRNA (Figure 4E), suggesting that S1PR1 might restrict lymphatic endothelial cell tube formation. This result is consistent with Geng X et al.’s report in which LEC-S1pr1 has been shown to prevent sprouting from quiescent lymphatic vessels (19). Our *in vitro* data suggested that the inhibitory effect of S1pr1 on LEC tube formation and the enhancing effect of S1pr1 on LEC migration/proliferation might counteract each other’s effect on lymphatic vascular angiogenesis, which might explain that no difference in lymphangiogenesis in post-MI myocardium between *WT* and *Lyve1-Cre-S1pr1*^flox/wt^** mice. This suggests that the effect of LEC-S1pr1 on post-MI cardiac remodeling was mediated by non-lymphangiogenic mechanisms.

### The reduced expression of LEC-S1pr1 retards macrophage clearance in injured hearts

**FIGURE 5:**
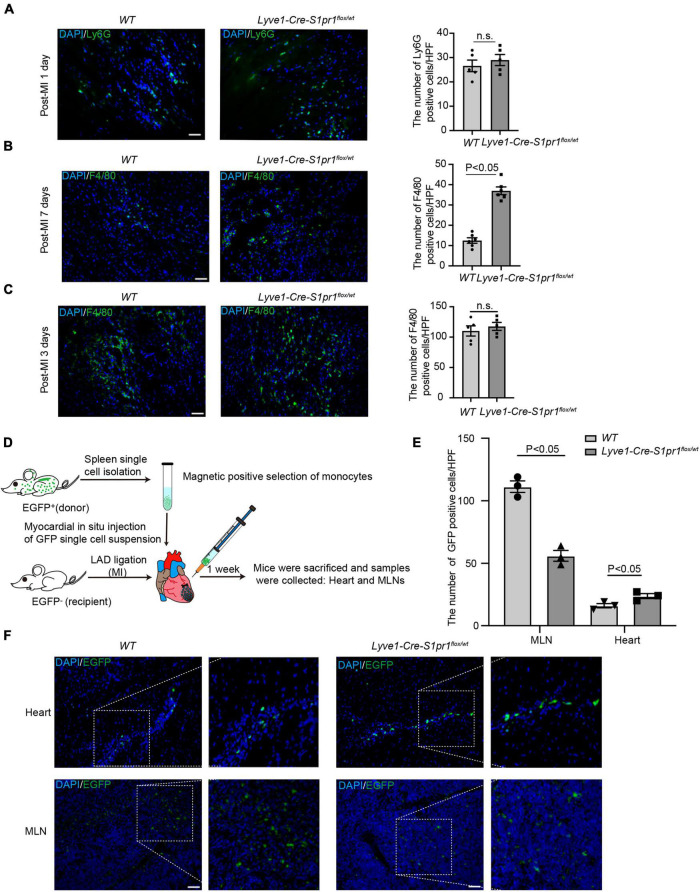
The reduced expression of LEC-S1pr1 retards macrophage clearance in injured hearts. (**A**) Representative images of immunostaining of Ly6G (neutrophil marker) from hearts in *WT* and *Lyve1-Cre-S1pr1*^flox/wt^** at 1 day after MI, with quantification of the number of neutrophils in infarct zone of myocardium of the indicated groups (*n* = 5). **(B)** Representative images of immunostaining of F4/80 (macrophage marker) within infarct zone of hearts in *WT* and *Lyve1-Cre-S1pr1*^flox/wt^** at 7 days after MI, with quantification of the number of macrophages in myocardium of the indicated groups (*n* = 6). **(C)** Representative images of immunostaining of F4/80 (macrophage marker) from hearts in *WT* and *Lyve1-Cre-S1pr1*^flox/wt^** at 3 days after MI, with quantification of the number of macrophages in infarct zone of myocardium of the indicated groups (*n* = 5). **(D)** Schematic diagram of the adoptive cell transfer approach. EGFP transgenic mice were used as splenic EGFP^+^ monocyte donors, and recipient *WT* or *Lyve1-Cre-S1pr1*^flox/wt^** adult mice received donor EGFP^+^ monocytes via intramyocardial delivery at the time of LAD ligation and EGFP donor monocyte trafficking was monitored in post-MI hearts and their draining MLNs (*n* = 3). **(E,F)** Representative fluorescence images of hearts and draining MLNs in *WT* and *Lyve1-Cre-S1pr1*^flox/wt^** at 7 days after MI, with quantification of the number of EGFP^+^ macrophages in the myocardium of the indicated groups. Less donor GFP + macrophages were detected in draining MLNs of *Lyve1-Cre-S1pr1*^flox/wt^** mice compared with *WT* mice, while more donor EGFP + macrophages in hearts of *Lyve1-Cre-S1pr1*^flox/wt^** mice (*n* = 3). Scale bars for **(A–C,F)**, 50 μm. Data are mean ± S.E.M. n.s., no statistical significance.

It has been shown that lymphatic vessels regulated the clearance of the infiltrated leukocytes in post-MI myocardium, and therefore influenced cardiac acute inflammatory responses and heart injury after MI (13). We first compared the number of infiltrating leukocytes in post-MI hearts between *Lyve1-Cre-S1pr1*^flox/wt^** mice and WT littermates. Although no difference was observed in the number of neutrophils (Figure 5A) in infarct zone of myocardium at 1 day after MI in *Lyve1-Cre-S1pr1*^flox/wt^** mice compared with WT littermates, a significant increase was detected in the number of macrophages in the infarct zone of hearts at 7 days after MI in *Lyve1-Cre-S1pr1*^flox/wt^** mice (Figure 5B). To further investigate whether *Lyve1-Cre-S1pr1*^flox/wt^** mice influenced cardiac resident macrophage after myocardial infarction, we performed co-immunostaining of CCR2 and F4/80 in post-MI hearts. Our results showed that there was no difference in the number of cardiac resident macrophages (CCR2^–^F4/80^+^) in the peri-infarct zone between *Lyve1-Cre-S1pr1*^flox/wt^** mice and *WT* littermates (Supplementary Figure 4A), suggesting that cardiac resident macrophages may not be influenced by *Lyve1-Cre-S1pr1*^flox/wt^** mice. In the infarct zone, there were minimal resident macrophages in infarct zone 3 days post-MI in both *Lyve1-Cre-S1pr1*^flox/wt^** mice and *WT* littermates (Supplementary Figure 4B). These results were well consistent with previous investigations which reported that resident cardiac macrophages were lost within infarct myocardium and that resident cardiac macrophages accounted only 2–5% of the total cardiac macrophages within the infarct zone during the first a few weeks post-infarct (23). To test whether LEC-S1pr1 influences the infiltration of macrophages and/or the clearance of macrophages in the infarct zone of post-MI hearts, we first examined whether circulating monocyte subpopulation was altered in *Lyve1-Cre-S1pr1*^flox/wt^** mice. Our cytometric analysis showed that *Lyve1-Cre-S1pr1*^flox/wt^** mice displayed similar monocyte subpopulations including CD11b^+^Ly6c^high^ and CD11b^+^Ly6c^ow^ as *WT* mice (Supplementary Figure 5) indicating that circulating monocyte/macrophage profile might not be influenced by heterozygous deletion of S1pr1 in Lyve1-Cre-expressing cells. We next enumerated infiltrated macrophages in myocardium at 3 days after MI, since those infiltrated macrophages reach a peak in the infarct zone of injured myocardium at 3 days after MI and that macrophage clearance is not evident until 7 days after MI following the establishment of an extensive lymphatic network draining the infarcted area (13). Our data showed that the number of macrophages in the infarct zone of *Lyve1-Cre-S1pr1*^flox/wt^** mice was as similar as WT mice at 3 days after MI, compared with *WT* mice (Figure 5C). These results suggested that a reduced expression of LEC-S1pr1 didn’t alter the infiltration of macrophages in an early inflammatory phase of MI. We next investigated whether LEC-S1pr1 might affect macrophage clearance in the later phase of MI. It has been shown that MLN as the secondary lymphatic organ serving the heart and that infiltrating macrophages in myocardium were transported via afferent cardiac lymphatics into draining mediastinal lymph nodes (MLNs). To investigate cardiac immune cell trafficking to MLNs, we employed adoptive cell transfer using ubiquitous EGFP expressing transgenic mice. Specifically, splenic EGFP + monocytes were isolated and transferred to a recipient *Lyve1-Cre-S1pr1*^flox/wt^** mice or *WT* littermates, via intramyocardial delivery, at the time of coronary artery ligation (Figure 5D). Less EGFP^+^ cells were detected in draining MLNs of *Lyve1-Cre-S1pr1*^flox/wt^** mice compared with *WT* mice, while more EGFP^+^ cells in hearts of *Lyve1-Cre-S1pr1*^flox/wt^** mice on day 7 following MI (Figures 5E,F). These data demonstrated that the reduced expression of LEC-S1pr1 didn’t influence the infiltration of macrophages in an early inflammatory phase of MI, but significantly affected macrophage clearance in the later phase of MI via afferent cardiac lymphatics, and thus aggravated post-MI cardiac acute inflammatory responses and worsened pathological cardiac remodeling.

### LEC-S1pr1 regulates macrophage trafficking via ERK/CCL2 signaling pathway

**FIGURE 6:**
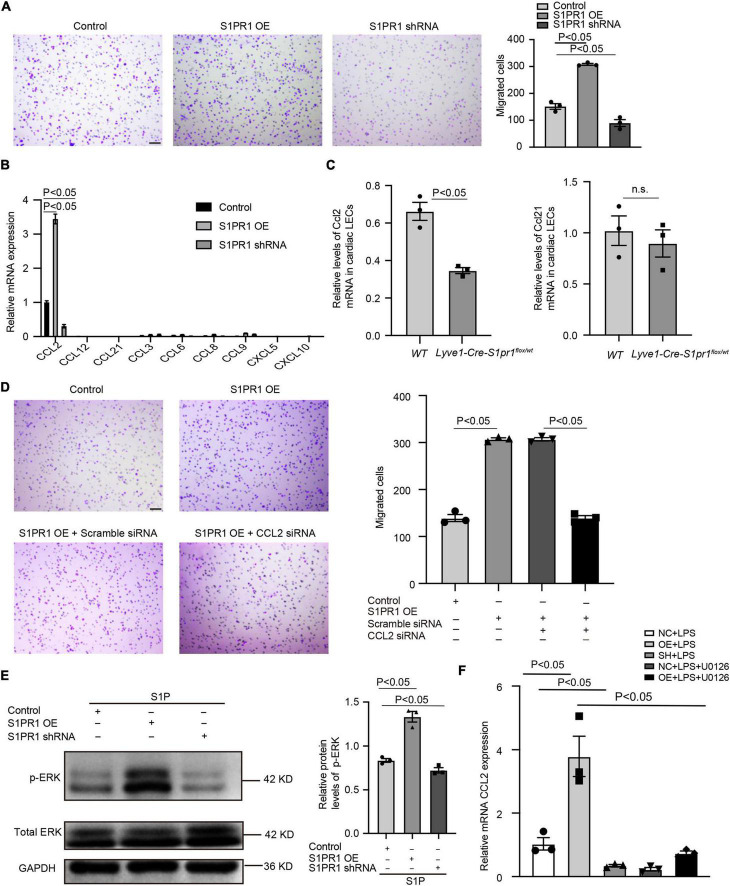
LEC-S1pr1 regulates macrophage trafficking via ERK/CCL2 signaling pathway. **(A)** The representative images of transwell cell migration assay of THP-1 cell lines, which were co-cultured with condition medium obtained from LECs of the indicated groups, with their quantification of migrated cells (*n* = 3). **(B)** RT-qPCR analysis showed the relative mRNA expression levels of different chemokines in the indicated groups (*n* = 3). **(C)** RT-qPCR analysis showed the relative Ccl2 and Ccl21 mRNA expression levels in LECs from hearts of the indicated groups at 7 days after MI (*n* = 3). **(D)** The representative images of Boyden chamber assay of THP-1 cell lines, which were co-cultured with the conditioned medium obtained from LECs of the indicated groups, with their quantification of migrated THP-1 (*n* = 3). **(E)** Western blotting analysis of ERK activation status in LECs treated with S1P in the indicated groups with their quantification of the ratio of p-ERK/total ERK (*n* = 3). **(F)** RT-qPCR showed the CCL2 mRNA expression of the indicated groups (*n* = 3). U0126, ERK inhibitor. Scale bars for **(A,C)** 100 μm. Data are mean ± S.E.M. n.s., no statistical significance.

**FIGURE 7:**
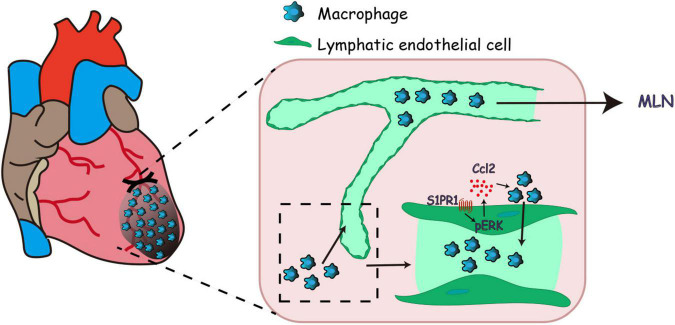
Working model of lymphatic endothelial S1pr1 enhances macrophage clearance via lymphatic system following myocardial infarction.

To further identify the molecular mechanism by which LEC-S1pr1 influences macrophage clearance, we tested the effect of HLEC-conditioned medium on the migration of macrophages *in vitro*. Our data showed that the conditioned medium from S1PR1-overexpressing HLEC culture significantly enhanced macrophage transmigration *in vitro* (Figure 6A), suggesting that LECs might secrete chemokines via S1P/S1pr1 signaling to recruit macrophages and boost their migration in a paracrine manner. We next investigated whether LEC-S1pr1 regulated key chemokines which control macrophage chemotactic migration. Among various chemokines, CCL2 was the major chemokine expressed in HLECs, and significantly higher expression levels of CCL2 were observed in S1PR1-overexpressing HLECs, while lower expression in S1PR1-silencing HLECs (Figure 6B). As expected, we detected lower expression levels of Ccl2 in LECs from heats of *Lyve1-Cre-S1pr1*^flox/wt^** mice at 7 days following MI, in comparison with *WT* littermates (Figure 6C); however, the main lymphatic chemokine, Ccl21, was not altered in LECs of *Lyve1-Cre-S1pr1*^flox/wt^** mice (Figure 6C). Our results further showed that inhibition of CCL2 expression by siRNA blocked the enhancing effect of HLEC-S1PR1 overexpression on macrophage transmigration (Figure 6D). To further investigate which signaling pathway was involved in the regulation of LEC-S1PR1 on CCL2 expression, we investigated the influence of LEC-S1PR1 on the ERK signaling pathway which has been widely reported to be activated by S1PR1 in various cells (3). Our western-blot analysis showed that the active levels of ERK were significantly higher in S1PR1-overexpressing HLECs, while lower in S1PR1-silencing HLECs, suggesting S1PR1 up-regulated ERK activity in LECs (Figure 6E). To further investigate whether the effects of LEC-S1pr1 on CCL2 expression are dependent on ERK signaling pathway, we treated LECs with ERK inhibitor, U0126, in S1PR1-overexpressing LECs *in vitro*. The inhibition of the ERK signaling pathway reversed the up-regulated expression of CCL2 in S1PR1-overexpressing HLECs (Figure 6F). These results suggest that LEC-S1pr1 might regulate CCL2 expression via ERK signaling pathway. Taken together, our results demonstrate that LEC-S1pr1 might regulate macrophage recruitment and migration via the ERK-CCL2 pathway in a paracrine manner (Figure 7).

## Discussion

There are two vascular circulatory systems in mammals: one is the blood vasculature; the other is the lymphatic vasculature. It has been well documented that S1pr1 was essential for maintaining vascular stability under both physiological and pathophysiological conditions (24). However, the effect of S1pr1 on lymphatic vasculature has been just recently reported by Geng X et al. (19). Geng X et al. showed that loss of S1pr1 in LECs induced lymphatic vascular hyper-sprouting and hyperbranching in a Vegfr3-dependent manner *in vivo* and that S1pr1 controlled lymphatic vessel maturation via RhoA signaling pathway by promoting membrane localization of the tight junction molecule claudin-5 (19). S1pr1 dampens LSS/VEGF-C signaling, suggesting that S1pr1 plays an important role in lymphatic vascular hemostasis during embryo development (19). However, whether LEC-S1pr1 is involved in the regulation of pathological ventricular remodeling is unknown. In this report, our data showed that the expression of S1pr1 in LECs was significantly reduced in hearts after myocardial infarction. Our further *in vivo* experiments showed that a reduced expression of S1pr1 in LECs deteriorated pathological ventricular remodeling and worsened cardiac dysfunction after myocardial infarction, suggesting that LEC-S1pr1 exerts a key role in improving post-MI cardiac remodeling and functions.

The lymphatic vasculature is a unidirectional conduit with a range of functions, including maintenance of interstitial fluid balance and immune cell trafficking (18). After heart injury, leukocyte transport determines the extent of cardiac injury and influences the following myocardial healing (13). Vieira JM et al. reported that cardiac lymphatic vessels promoted leukocyte clearance from injured hearts, resulting in dampening cardiac injury and improving cardiac healing (13). They suggested that VEGFC enhanced post-MI lymphangiogenesis and thus promoted leukocyte exit from injured hearts (13). However, one recent report reported that lymphangiogenesis may not contribute to the beneficial effect of VEGFC on cardiac repair after MI (25). The molecular mechanism by which cardiac lymphatic vasculature regulates the egress of immune cells from hearts and its role in cardiac repair after heart injury are not yet fully understood. Herein, we identified that LEC-S1pr1 was critical for the clearance of infiltrating macrophages in the myocardium, since a reduction of LEC-S1pr1 significantly reduced macrophage exists from the post-MI hearts. Our further data suggested this effect of LEC-S1pr1 on macrophage clearance from ischemic hearts might not be due to post-MI lymphangiogenesis, as demonstrated by a similar lymphatic vessel density in myocardium between *WT* and *S1pr1* transgenic mice.

It is known that the passage of immune cells from tissue interstitium toward afferent lymphatic vessels depends on chemotaxis (18). Previous investigations showed that lymphatic endothelium expressed and secreted various chemokines, including CCL2, CCL12, CCL5, CCL20, CXCL2, and CX3CL1 (fractalkine), which direct selective egress from tissues of leukocytes (26). We next asked whether LEC-S1pr1 regulated the expression of chemokines, which directs leukocytes toward LECs and enhanced their egress from injured hearts. Our data showed that LEC-S1pr1 influenced the expression of multiple chemokines, including CCL2, CCL3, and CCL6. Among these cytokines, CCL2 is the major chemokine which was expressed and significantly regulated by S1pr1 signaling in LECs. It has been well documented that CCL2, monocyte chemoattractant protein-1 (MCP-1), played an essential role in myocardial pathology and was sharply upregulated in post-MI myocardium (27). *In vivo* animal studies have shown that CCL2 regulated monocytes/macrophages recruitment, activation, and polarization in the injured heart after MI, and thus influenced pathological ventricular remodeling and cardiac repair (27). Previous studies revealed that LECs expressed CCL2, suggesting that LEC-CCL2 is involved in the regulation of leukocyte chemotactic migration (28, 29). In consistent with previous reports (28, 29), we identified that CCL2 was expressed in LECs, and that LEC-expressing CCL2 was regulated by S1pr1. Previous studies showed that ERK signaling pathway was involved in the regulation of the expression of multiple genes, including CCL2 (17, 30, 31). In consistence with these reports (17, 30, 31), our data showed that S1pr1 enhanced the expression of CCL2 in LECs via the ERK signaling pathway.

Taken together, previous investigations indicated that S1pr1 tightly controls lymphatic vascular homeostasis during embryogenesis; however, the LEC-specific effects of S1pr1 on post-MI pathological ventricular remodeling are unknown. Using LEC-specific S1pr1 transgenic mice, we presented that the reduced expression of S1pr1 in LECs worsened post-MI ventricular remodeling and cardiac dysfunctions, providing strong *in vivo* evidence to support an essential role of LEC-S1pr1 in the regulation of cardiac repair and functions after MI. Mechanically, S1pr1 signaling activates ERK signaling pathway in LECs, and boosts CCL2 expressions, resulting in the recruitment of infiltrating macrophages in myocardium toward lymphatic endothelium and an enhancement in macrophage clearance from ischemic myocardium via afferent cardiac lymphatics, and consequently dampens post-MI inflammation and improves cardiac functions (Figure 7). Our study reveals a novel role of LEC-S1pr1 in the regulation of post-MI cardiac remodeling and functions, providing a potential therapy by modulation of LEC-S1pr1 to resolve cardiac inflammation and to improve cardiac functions after myocardial infarction.

## Data availability statement

The datasets presented in this study can be found in online repositories. The names of the repository/repositories and accession number(s) can be found below: BioProject, PRJNA818802.

## Ethics statement

The animal study was reviewed and approved by Tongji University Institutional Animal Care and Use Committee.

## Author contributions

SD and JW contributed to the conception and design of the study, and wrote the manuscript. QL performed experiments and acquire data. CZ, KZ, and YD quantify experimental data. All authors contributed to manuscript revision, read, and approved the submitted version.

## Supplementary material

Supplementary Figure 1 Flow cytometric analysis showed the purity of isolated cardiac LECs without monocyte/macrophage contamination. **(A)** The representative images of flow cytometric analysis of cardiac cells from post-MI hearts of WT mice before LECs separation. **(B)** The representative images of flow cytometric analysis of cardiac cells from post-MI hearts of WT mice after LECs separation. **(C)** The representative images of flow cytometric analysis of peripheral blood cells of WT mice by staining CD45 or CD11b or Ly6C antibody and its corresponding IgG control validated the optimization of flow cytometric analysis in our study.

Supplementary Figure 2 Relative mRNA expression levels in cardiac LECs of mice. **(A)** Relative mRNA expression levels of S1pr2 in cardiac LECs of mice at 3 days after sham or LAD operation (*n* = 6). **(B)** Relative mRNA expression levels of S1pr3 in cardiac LECs of mice at 3 days after sham or LAD operation (*n* = 6). **(C)** Relative mRNA expression levels of S1pr4 in cardiac LECs of mice at 3 days after sham or LAD operation (*n* = 6). Data are mean ± S.E.M. n.s., no statistical significance.

Supplementary Figure 3 The reduced expression of LEC-S1pr1 did not influence the proliferation of lymphatic endothelial cells after myocardial infarction. Representative images of immunostaining of Ki67 (cell proliferation marker) and Lyve-1 (LEC marker) from hearts in *WT* and *Lyve1-Cre-S1pr1*^flox/wt^** at 7 days after MI, with quantification of the number of proliferating lymphatic endothelial cells in myocardium of the indicated groups (*n* = 5). Scale bars, 50 μm. Data are mean ± S.E.M. n.s., no statistical significance.

Supplementary Figure 4 The reduced expression of LEC-S1pr1 did not influence the resident macrophages in peri-infarct zone after myocardial infarction. **(A)** Representative images of immunostaining of CCR2 and F/480 from hearts in *WT* and *Lyve1-Cre-S1pr1*^flox/wt^** at 3 days after MI, with quantification of the number of CCR2^–^F4/80^+^ cells (cardiac resident macrophages) in the peri-infarct zone of indicated groups (*n* = 5). **(B)** Representative images of immunostaining of CCR2 and F/480 from hearts in *WT* and *Lyve1-Cre-S1pr1*^flox/wt^** at 3 days after MI, with quantification of the number of CCR2^+^F4/80^+^ cells (the recruited macrophages from circulation) in the infarct zone of indicated groups (*n* = 5). Scale bars for **(A–B)**, 50 μm. Data are mean ± S.E.M. n.s., no statistical significance.

Supplementary Figure 5 The monocyte subpopulation profile in peripheral blood. The representative cytometric images of circulating CD11b^+^ly6C^high/low^ monocyte subpopulations with their quantification in *WT* and *Lyve1-Cre-S1pr1*^flox/wt^** mice.
