# Supplementary figures and images for: Lymphatic endothelial sphingosine 1-phosphate receptor 1 enhances macrophage clearance via lymphatic system following myocardial infarction
Source: Front Cardiovasc Med. 2022 Aug 8;9:872102. doi: 10.3389/fcvm.2022.872102 (PMC9393290; doi:10.3389/fcvm.2022.872102)

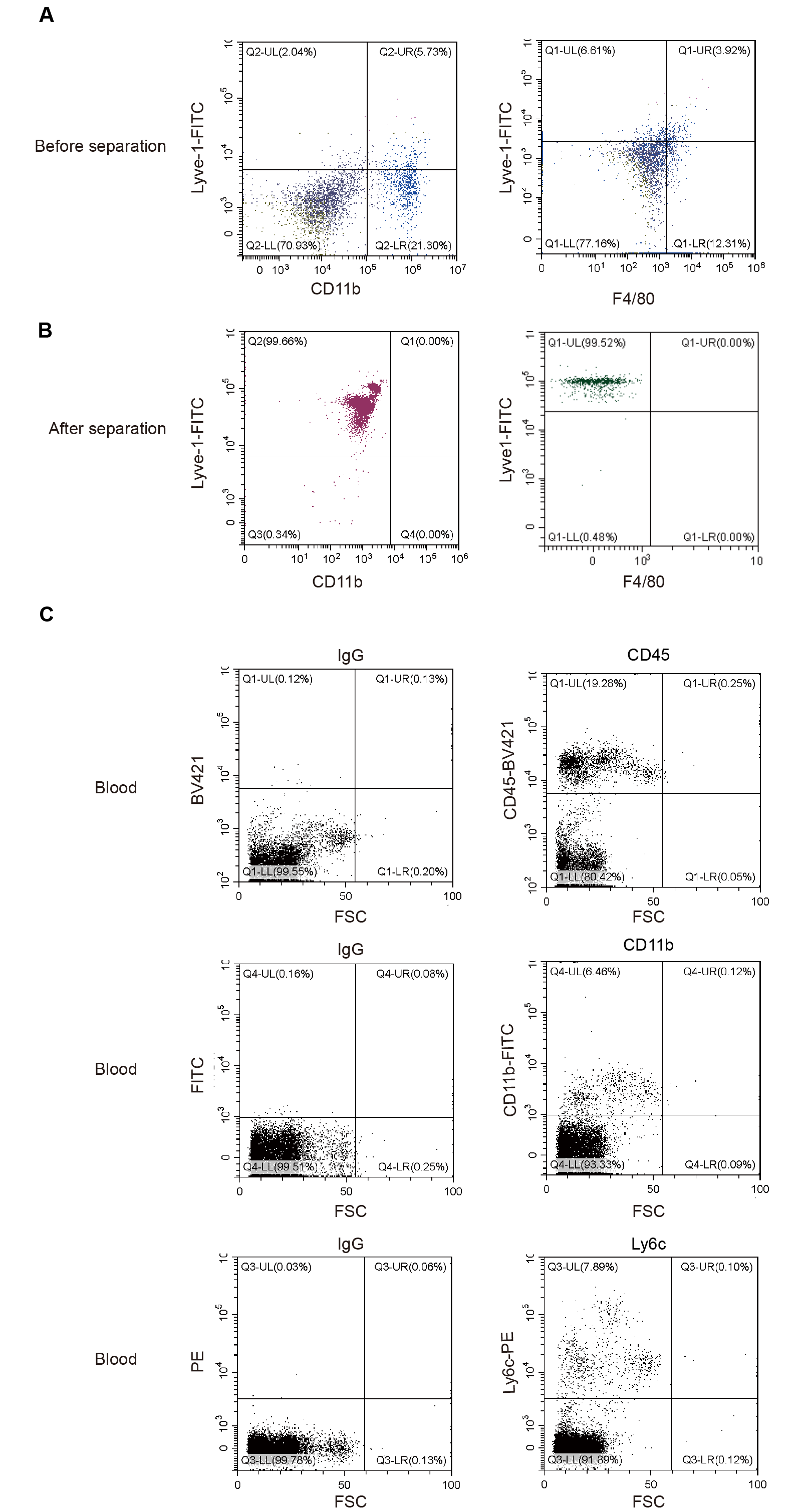

Supplement: Supplementary Figure 1 — Flow cytometric analysis showed the purity of isolated cardiac LECs without monocyte/macrophage contamination. (A) The representative images of flow cytometric analysis of cardiac cells from post-MI hearts of WT mice before LECs separation. (B) The representative images of flow cytometric analysis of cardiac cells from post-MI hearts of WT mice after LECs separation. (C) The representative images of flow cytometric analysis of peripheral blood cells of WT mice by staining CD45 or CD11b or Ly6C antibody and its corresponding IgG control validated the optimization of flow cytometric analysis in our study. [file Data_Sheet_1.zip › supp/figure-1.tif]

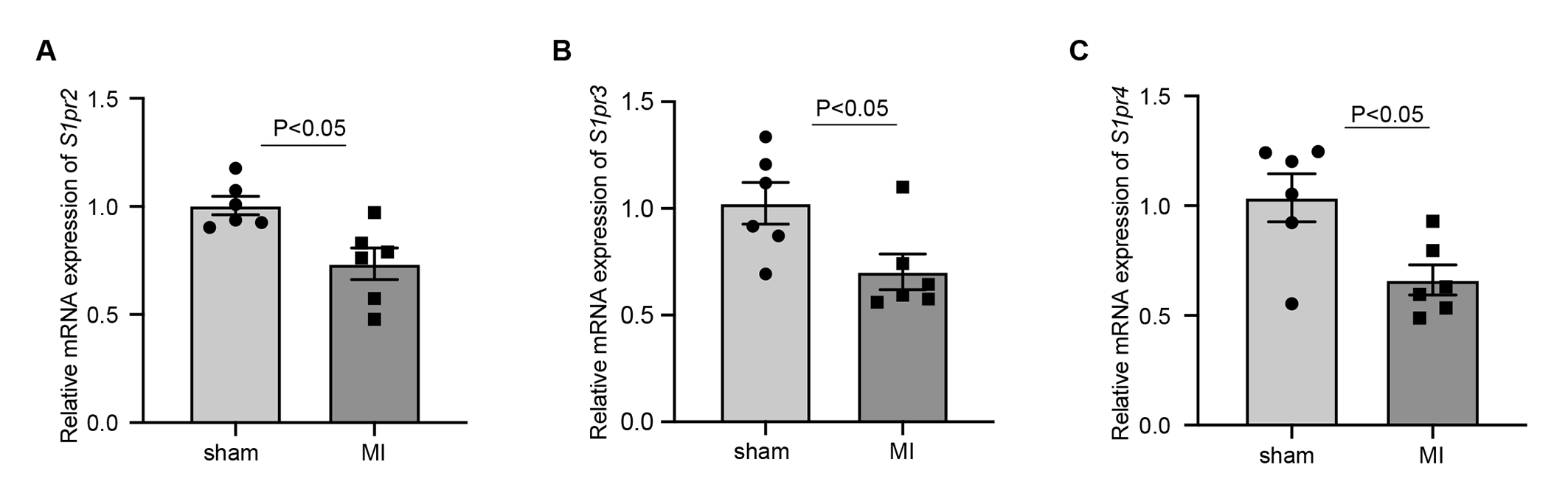

Supplement: Supplementary Figure 1 — Flow cytometric analysis showed the purity of isolated cardiac LECs without monocyte/macrophage contamination. (A) The representative images of flow cytometric analysis of cardiac cells from post-MI hearts of WT mice before LECs separation. (B) The representative images of flow cytometric analysis of cardiac cells from post-MI hearts of WT mice after LECs separation. (C) The representative images of flow cytometric analysis of peripheral blood cells of WT mice by staining CD45 or CD11b or Ly6C antibody and its corresponding IgG control validated the optimization of flow cytometric analysis in our study. [file Data_Sheet_1.zip › supp/figure-2.tif]

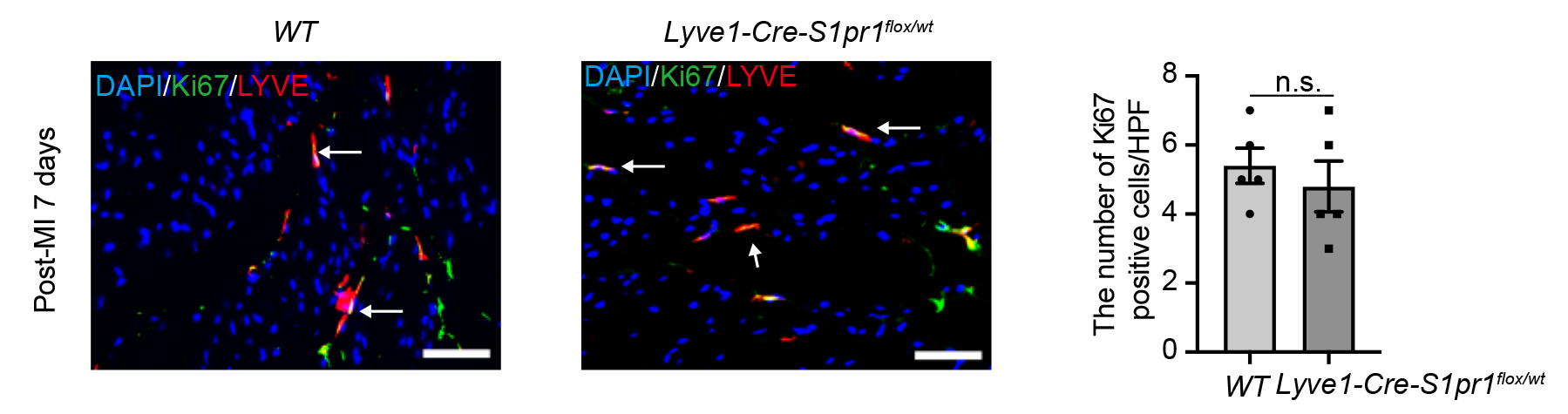

Supplement: Supplementary Figure 1 — Flow cytometric analysis showed the purity of isolated cardiac LECs without monocyte/macrophage contamination. (A) The representative images of flow cytometric analysis of cardiac cells from post-MI hearts of WT mice before LECs separation. (B) The representative images of flow cytometric analysis of cardiac cells from post-MI hearts of WT mice after LECs separation. (C) The representative images of flow cytometric analysis of peripheral blood cells of WT mice by staining CD45 or CD11b or Ly6C antibody and its corresponding IgG control validated the optimization of flow cytometric analysis in our study. [file Data_Sheet_1.zip › supp/figure-3.tif]

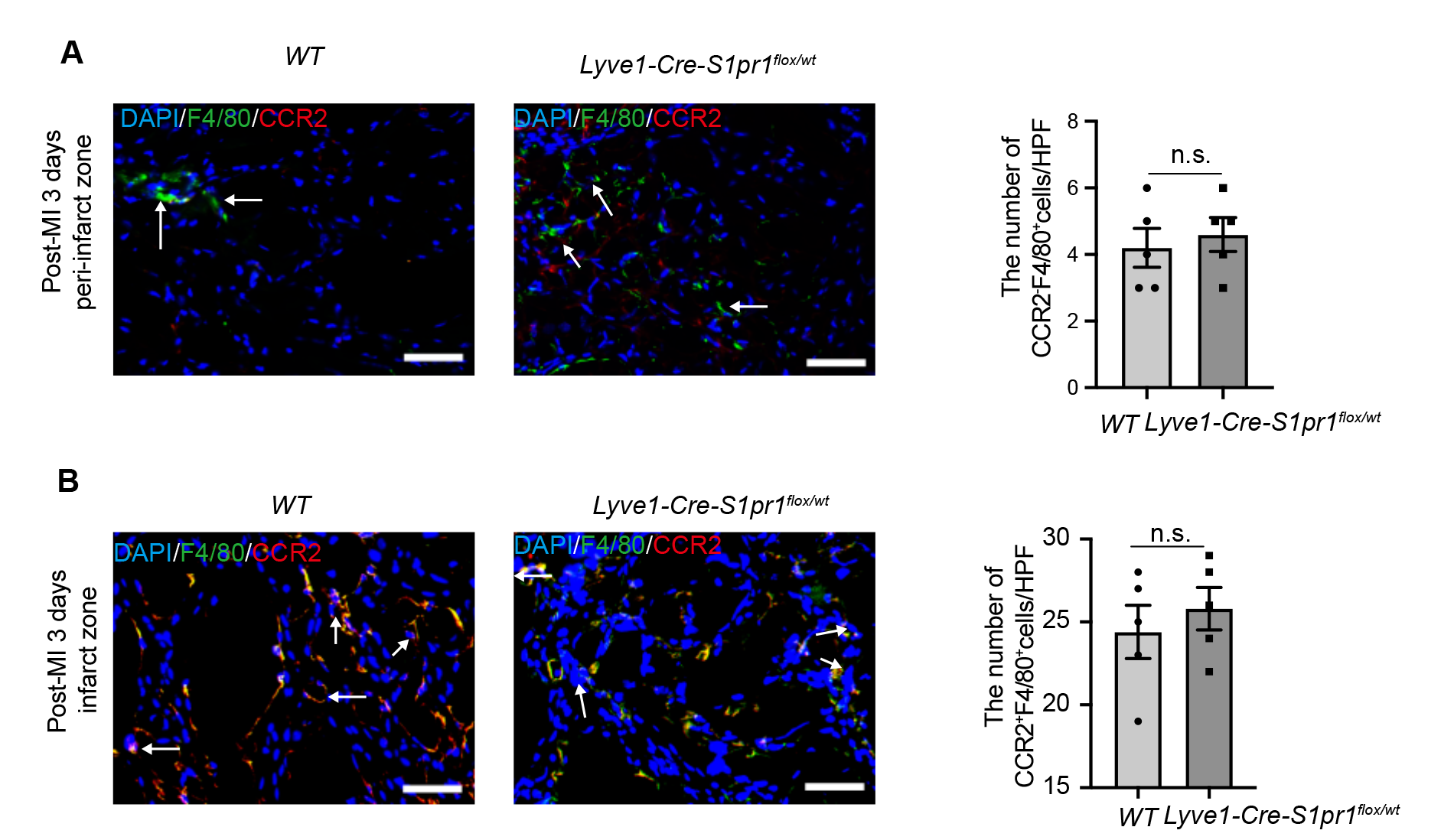

Supplement: Supplementary Figure 1 — Flow cytometric analysis showed the purity of isolated cardiac LECs without monocyte/macrophage contamination. (A) The representative images of flow cytometric analysis of cardiac cells from post-MI hearts of WT mice before LECs separation. (B) The representative images of flow cytometric analysis of cardiac cells from post-MI hearts of WT mice after LECs separation. (C) The representative images of flow cytometric analysis of peripheral blood cells of WT mice by staining CD45 or CD11b or Ly6C antibody and its corresponding IgG control validated the optimization of flow cytometric analysis in our study. [file Data_Sheet_1.zip › supp/figure-4.tif]

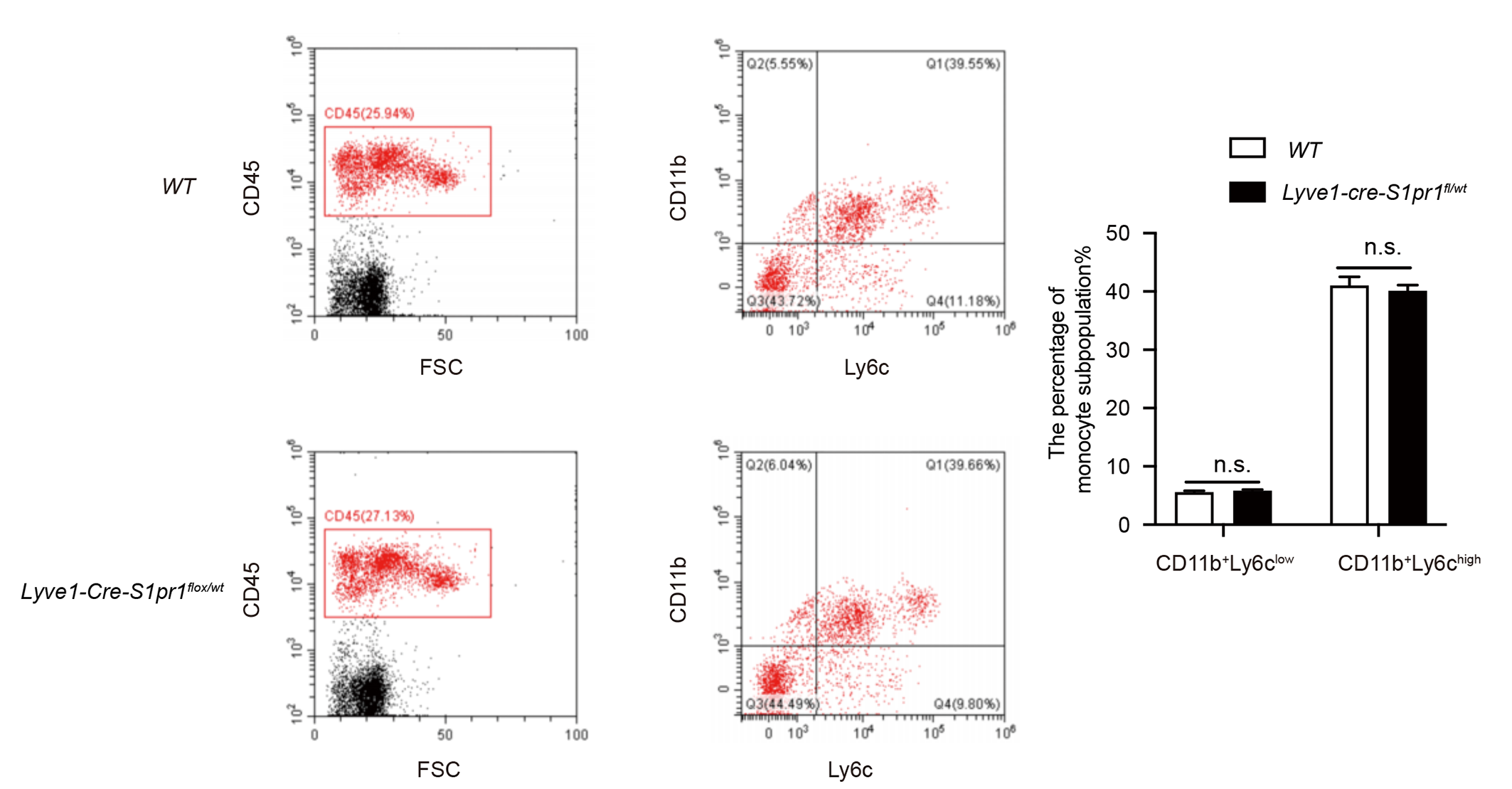

Supplement: Supplementary Figure 1 — Flow cytometric analysis showed the purity of isolated cardiac LECs without monocyte/macrophage contamination. (A) The representative images of flow cytometric analysis of cardiac cells from post-MI hearts of WT mice before LECs separation. (B) The representative images of flow cytometric analysis of cardiac cells from post-MI hearts of WT mice after LECs separation. (C) The representative images of flow cytometric analysis of peripheral blood cells of WT mice by staining CD45 or CD11b or Ly6C antibody and its corresponding IgG control validated the optimization of flow cytometric analysis in our study. [file Data_Sheet_1.zip › supp/figure-5.tif]
